# In Vivo Analysis of the Biocompatibility and Immune Response of Jellyfish Collagen Scaffolds and its Suitability for Bone Regeneration

**DOI:** 10.3390/ijms21124518

**Published:** 2020-06-25

**Authors:** Iris Flaig, Milena Radenković, Stevo Najman, Annica Pröhl, Ole Jung, Mike Barbeck

**Affiliations:** 1 BerlinAnalytix GmbH, 12109 Berlin, Germany; iris.flaig@me.com (I.F.); annica.proehl@berlinanalytix.com (A.P.); 2 Department for Cell and Tissue Engineering, Faculty of Medicine, University of Niš, 18108 Niš, Serbia; milena.radenkovic@pmf.edu.rs; 3 Department for Cell and Tissue Engineering and Department of Biology and Human Genetics, Faculty of Medicine, University of Niš, 18108 Niš, Serbia; stevo.najman@medfak.ni.ac.rs; 4 Clinic and Policlinic for Dermatology and Venereology, University Medical Center Rostock, 18057 Rostock, Germany; ole.tiberius.jung@gmail.com

**Keywords:** jellyfish collagen, macrophage, bone regeneration, biomaterial integration, immune response, tissue regeneration

## Abstract

Jellyfish collagen, which can be defined as “collagen type 0” due to its homogeneity to the mammalian types I, II, III, V, and IX and its batch-to-batch consistent producibility, is of special interest for different medical applications related to (bone) tissue regeneration as an alternative to mammalian collagen-based biomaterials. However, no in vivo studies regarding the induction of M1- and M2-macrophages and their time-dependent ration as well as the analysis of the bone regeneration capacity of jellyfish collagen scaffolds have been conducted until now. Thus, the goal of this study was to determine the nature of the immune response to jellyfish collagen scaffolds and their bone healing capacities. Two in vivo studies using established implantation models, i.e., the subcutaneous and the calvarian implantation model in Wistar rats, were conducted. Furthermore, specialized histological, histopathological, and histomorphometrical methods have been used. As a control biomaterial, a collagen scaffold, originating from porcine pericardium, which has already been stated as biocompatible, was used for the subcutaneous study. The results of the present study show that jellyfish collagen scaffolds are nearly completely resorbed until day 60 post implantation by stepwise integration within the subcutaneous connective tissue mediated mainly by macrophages and single multinucleated giant cells. Interestingly, the degradation process ended in a vessel rich connective tissue that is understood to be an optimal basis for tissue regeneration. The study results showed an overall weaker immune response to jellyfish collagen than to porcine pericardium matrices by the induction of significantly lower numbers of macrophages together with a more balanced occurrence of M1- and M2-macrophages. However, both collagen-based biomaterials induced balanced numbers of both macrophage subtypes, which supports their good biocompatibility. Moreover, the histomorphometrical results for the calvarial implantation of the jellyfish scaffolds revealed an average of 46.20% de novo bone formation at day 60, which was significantly higher compared to the control group. Thereby, the jellyfish collagen scaffolds induced also significantly higher numbers of anti-inflammatory macrophages within the bony implantation beds. Altogether, the results show that the jellyfish collagen scaffolds allowed for a directed integration behavior, which is assumed to be in accordance with the concept of Guided Bone Regeneration (GBR). Furthermore, the jellyfish collagen scaffolds induced a long-term anti-inflammatory macrophage response and an optimal vascularization pattern within their implant beds, thus showing excellent biocompatibility and (bone) tissue healing properties.

## 1. Introduction

Biomaterials such as bone substitute materials (BSM) should support the regeneration process without adversely affecting the living organism and its components. Furthermore, they should allow for an adapted integration behavior needed for tissue regeneration. In the context of bone regeneration, a material should trigger directed growth of new bone at sites with insufficient volumes or dimensions of bone, following the concept of Guided Bone Regeneration (GBR). The most common need for GBR is the augmentation of bone for successful dental implant placement [1]. Traditionally, calcium phosphate-based materials (CaM) such as hydroxyapatite or beta-tricalcium phosphate are used as basic compounds for BSM due to their chemical similarity to the extracellular calcified bone matrix [2,3,4]. Moreover, different materials are able to support the bone regeneration process such as extracellular matrix proteins for use alone or in combination with CaM [5,6]. An example of such extracellular matrix proteins that can be used in combination with CaM include the biopolymer collagen. Collagen makes up a major protein component of the extra cellular matrix, which is secreted by cell types such as fibroblasts or osteoblasts [7,8]. Collagen’s proven biocompatibility, biodegradability, low immunogenicity, and cell-adhesive properties [9], but also the ability to provide appropriate signals for directing the cellular processes leading to tissue formation [10], makes this polymer the most frequently utilized material for tissue regeneration and tissue engineering. In the context of bone tissue regeneration, collagen is of special interest as basis for BSM as it is well known as a regulator of osteoblastic differentiation and cellular ingrowth, as well as promoting the secretion of bone matrix by differentiated osteoblasts [11,12]. Furthermore, collagen scaffolds have shown to promote the process of angiogenesis by interaction with (pre-) endothelial cells triggering vascularization of the implant bed, an important underlying factor of tissue regeneration [13,14]. Vascularization is one of the great challenges facing tissue engineering, as a vascular network capable of distributing oxygen and other nutrients is required to support cell survival [15,16].

Natural collagen can be extracted from a variety of organisms traditionally sourced for medical applications by extracting from bovine or porcine sources. There are growing regulatory concerns around the continued use of mammalian collagens as they are considered a pathological risk for transmitted diseases such as avian and swine influenzas, and tooth-and-mouth disease [17]. Collagen of bovine origin is, in particular, associated as high risk in terms of bovine spongiform encephalopathy (BSE) and transmissible spongiform encephalopathy (TSE), as well as potential viral vectors that could be transmissible to humans [18]. Furthermore, the purification of these proteins to reduce the risk of transmitted disease without structure alteration is very challenging [17]. It has also been reported that different mammalian collagen-based materials induce pro-inflammatory tissue responses due to their purification processes [19,20,21]. In addition, mammalian collagen, especially porcine origin, is increasingly rejected for religious or ethical reasons [9].

Hence, collagen extracted from a non-mammalian origin, seems to be an interesting alternative to address these issues associated with existing collagen-based biomaterials. Interestingly, marine organisms present an attractive alternative, due to lack of BSE risk and potential viral vectors [18]. The isolation and characterization of collagen from different fish and jellyfish species has already been described and a variety of differences between these collagen types have been reported [9,22,23,24,25,26]. Moreover, the usability of marine collagens has already been analyzed and it has been shown that jellyfish collagen is non-toxic and induces a higher cell viability of fibroblasts and osteoblasts compared to bovine collagen [10,22]. Additionally, Addad and colleagues conducted a study that tested different Mediterranean jellyfish species in order to investigate the different methods of collagen purification [22]. It was concluded that the best collagen yield was obtained using *Rhizostoma pulmo* (*R. pulmo*) and furthermore upon biological analysis, the cytotoxicity of *R. pulmo* collagen was no different in comparison to mammalian collagen.

Further studies supporting the potential of jellyfish collagen’s biocompatibility have been conducted, which included cytotoxicity tests, measurements of pro-inflammatory cytokine secretion, antibody secretion, as well as the population change of immune cells after *in vivo* implantation [22]. In their study, Song and colleagues found that the number of dendritic cells (CD11c+) and macrophages (F4/80+) were similar in jellyfish collagen implanted into mice as to those of bovine- and gelatin-implanted mice [10]. Hence, they concluded that the jellyfish collagen scaffolds were able to induce a comparable immune response to that caused by bovine collagen or gelatin [10].

With regard to biocompatibility, it is known that every biomaterial induces an inflammatory response directly after its implantation, in which macrophages are involved as key players due to their expression of regulating pro- and anti-inflammatory cytokines [27,28,29]. A biomaterial’s inflammatory alignment depends on its physicochemical properties and also appears to be responsible for its regenerative functionality [27,29]. Macrophages that modulate the inflammatory response to a biomaterial are either divided into 2 phenotypes. The M2 phenotype is considered as anti-inflammatory and are involved in wound healing functions [30]. In contrast, so-called M1-macrophages are pro-inflammatory subtypes and their occurrence seems to be correlated with disruption of normal tissue homeostasis and impede vascular repair [27,28,29]. Thus, it is a beneficial property for a biomaterial to effect an “overall M2 tissue reaction” as this can facilitate a successful clinical application [31].

However, to date, no in vivo studies regarding the induction of M1- and M2-macrophages and their time-dependent ration as well as the analysis of the bone regeneration capacity of jellyfish scaffolds have been conducted until now. Thus, the goal of this study was to determine the nature of the immune response to jellyfish collagen scaffolds and their healing capacities. Two in vivo studies using established implantation models, i.e., the subcutaneous and the calvarian implantation model, in Wistar rats were conducted. Furthermore, specialized histological, histopathological, and histomorphometrical methods as previously described have been used. As a control biomaterial, an already commercially available collagenous scaffold, originating from porcine pericardium, was used for the subcutaneous study. This biomaterial has already been examined in several preclinical and clinical studies and had been assessed as biocompatible [32,33,34].

## 2. Results

### 2.1. Results of the Histological Analyses

#### 2.1.1. Histological Analysis of the Subcutaneous Implants

**Figure 1:**
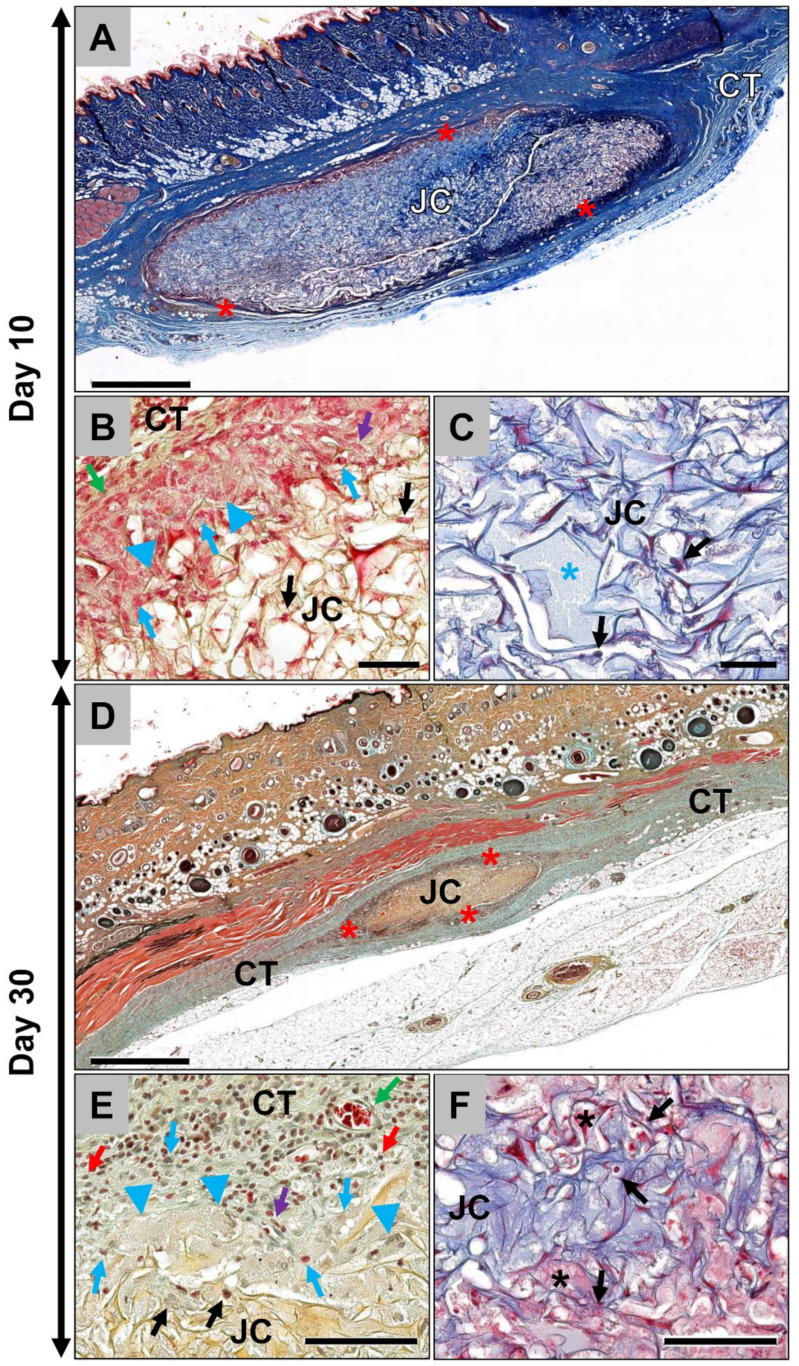
Histological images from the implantation beds of the analyzed jellyfish collagen scaffolds (JC) within the subcutaneous connective tissue (CT). (**A**) The jellyfish collagen scaffold (JC) has a distinct oval shape and a visible thin surface cell layer (red asterisks) at 10 days *post implantationem* (“total scan”, Masson’s Trichrome-staining, 10× magnification, scale bar = 1mm). (**B**) At the material surface, a layer composed of mononuclear cells that were mainly macrophages (blue arrows) and fibroblasts (purple arrow) as well as some single multinucleated giant cells (blue arrowheads) in direct neighborhood to small blood vessels (green arrow) were observable. Only a few mononuclear cells of the monocyte/macrophage line penetrated the outer regions of the scaffolds (black arrows) (Movat’s Pentachome-staining, 20× magnification, scale bar = 50 µm). (**C**) Only a very small number of mononuclear cells (black arrows), i.e., monocytes/macrophages, were present in the center of the scaffolds and most of the collagen fiber interspaces were filled with fibrin (blue asterisk) at this early study time point (Masson’s Trichrome-staining, 20× magnification, scale bar = 50 µm). (**D**) At day 30, the jellyfish collagen scaffolds (JC) were still observable within the subcutaneous implantation beds and were significantly reduced. Furthermore, a cell layer (red asterisks) was present at the material surfaces (Movat’s Pentachrome-staining, 10× magnification, scale bar = 1mm). (**E**) At the scaffold surface, mainly macrophages (blue arrows) and multinucleated giant cells (blue arrowheads) beside single fibroblasts (purple arrow) and lymphocytes (red arrows) in combination with blood vessels (green arrow) were detectable at this time point. Still, only single mononuclear cells were found within the outer regions of the scaffolds (black arrows) (Movat’s Pentachome-staining, 20× magnification, scale bar = 100 µm). (**F**) At this time point, also single mononuclear cells (black arrows) and provisional matrix (black asterisks) were found within the scaffold centers (Masson’s Trichrome-staining -staining, 20× magnification, scale bar = 100 µm).

The results of the histological analysis showed that the jellyfish collagen scaffolds (Jellagen^®^-3D scaffolds from Jellagen^®^ Ltd., Cardiff, UK) were detectable within the subcutaneous connective tissue at day 10 *post implantationem* surrounded by a thin wall of interacting cells (Figure 1A). A more detailed analysis revealed that mainly macrophages and some single multinucleated giant cells beside single fibroblasts and low numbers of granulocytes were observable at the outer scaffold surfaces (Figure 1B). Only low numbers of cells penetrated the scaffolds at this early post-implantation time point (Figure 1B). Thus, the inner core of the scaffolds was nearly cell-free but single mononuclear cells of the monocyte/macrophage-line were also detectable within this implant region at day 10 *post implantationem* (Figure 1C). At this early time point, no vessels were found within the scaffolds but directly located within the adherent connective tissue surrounding the scaffolds (Figure 1B).

At day 30 post implantation, the jellyfish scaffolds were still detectable within the subcutaneous connective tissue surrounded by the thin cell-rich layer (Figure 1D). At this time point, it was clearly visible that the volume of the scaffolds had decreased (Figure 1D). At the scaffold surface, mainly macrophages and multinucleated giant cells were observed in concert with single lymphocytes and fibroblasts (Figure 1E). In addition, at day 30, the inner core of the scaffolds was nearly free of cells as only very few mononuclear cells of the macrophage line were observable within this implant region (Figure 1F). 

**Figure 2:**
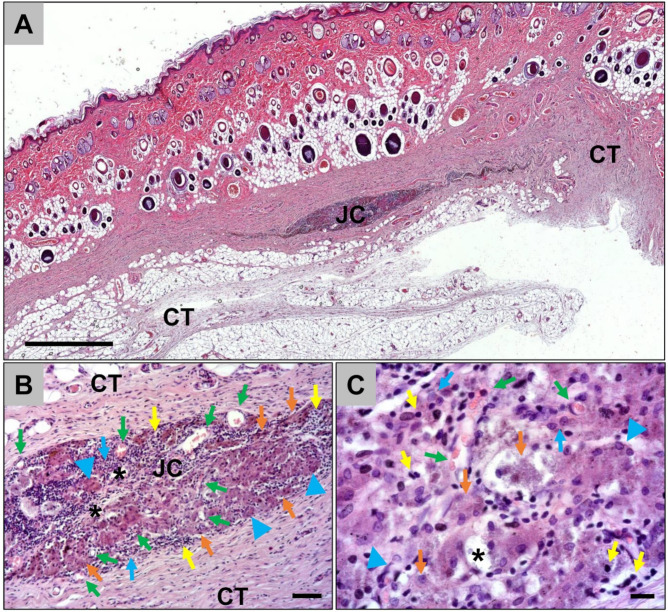
Representative histological images from the implantation beds of the jellyfish scaffolds within the subcutaneous connective tissue (CT) at day 60 *post implantationem*. (**A**) The remnants of the jellyfish collagen scaffold (JC) were observable at 60 days *post implantationem* (“total scan”, HE-staining, 10× magnification, scale bar = 1mm). (**B**) and (**C**) The remnants of the jellyfish collagen scaffolds (black asterisks) were surrounded by a cell- and vessel-rich (green arrows) granulation tissue at this time point. Beside macrophages (blue arrows) and multinucleated giant cells (blue arrowheads), high numbers of lymphocytes (yellow arrows) were detected. Interestingly, many macrophages showed signs of an increased cytosolic volume (orange arrows), indicating their phagocytic activity were observable (HE-stainings, A: 10× magnification, scalebar = 50 µm, B: 40× magnification, scale bar = 10 µm).

At day 60 post implantation, only a few remnants of the jellyfish collagen scaffolds were found within the subcutaneous implantation beds (Figure 2). The remnants were surrounded by a cell- and vessel-rich granulation tissue mainly composed of macrophages and lymphocytes together with single multinucleated giant cells (Figure 2). Many of the macrophages within the implantation beds showed signs of an increased cytosolic volume, indicating their phagocytic activity (Figure 2).

**Figure 3:**
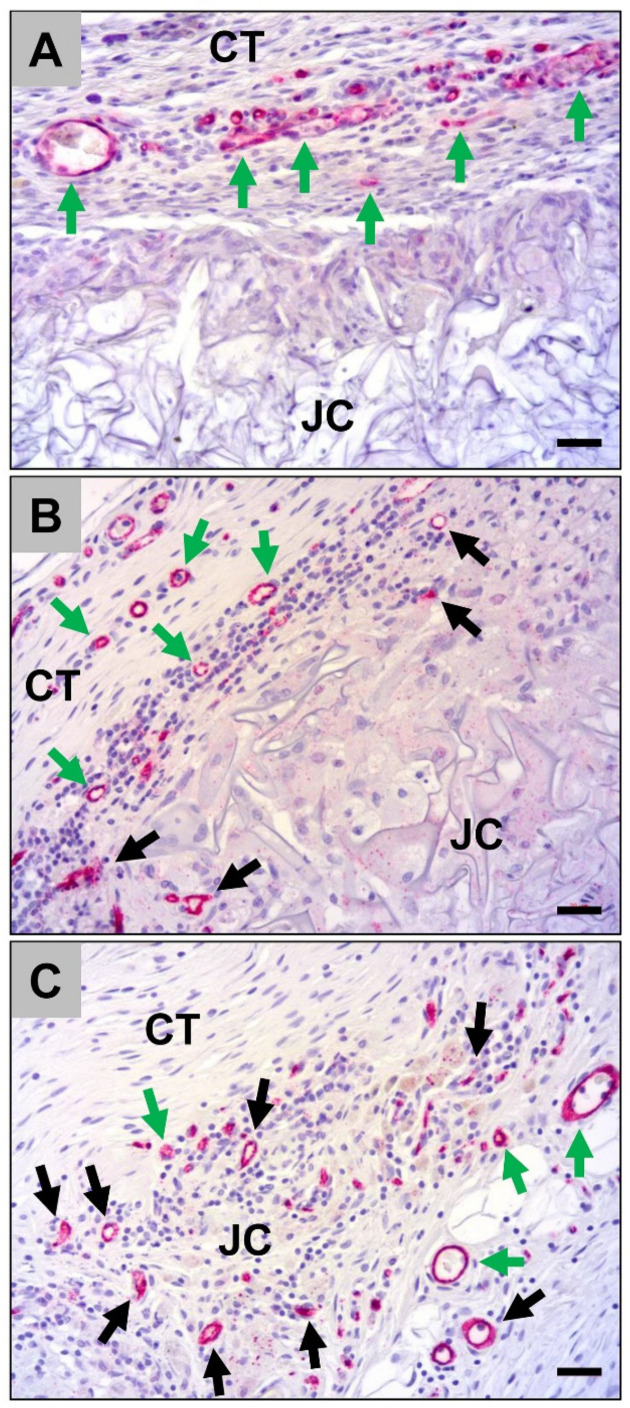
Representative histological images of the vascularization pattern of the jellyfish scaffolds (JC) within the subcutaneous connective tissue (CT) at day 10 (**A**), day 30 (**B**), and day 60 *post implantationem* (**C**). Green arrows = vessels within the surrounding connective tissue, black arrows = vessels within the jellyfish collagen scaffolds (CD31-immunostainings, 20× magnifications, scalebars = 20 µm).

Histological analysis of the implant bed vascularization showed that a high number of small and medium-sized blood vessels were located within the adherent connective tissue near to the scaffold surface at day 10 *post implantationem* (Figure 3A). At day 30 *post implantationem*, high numbers of vessels were still observed within direct neighborhood of the scaffold surfaces and an ingrowth of moderate numbers of vessels within the jellyfish collagen scaffolds was also visible (Figure 3B). At day 60 *post implantationem*, high numbers of small vessels were observable within the granulation tissue, which was detectable within the implantation beds of the jellyfish collagen scaffolds, while medium-sized vessels were predominantly found within the neighbored connective tissue (Figure 3C). 

**Figure 4:**
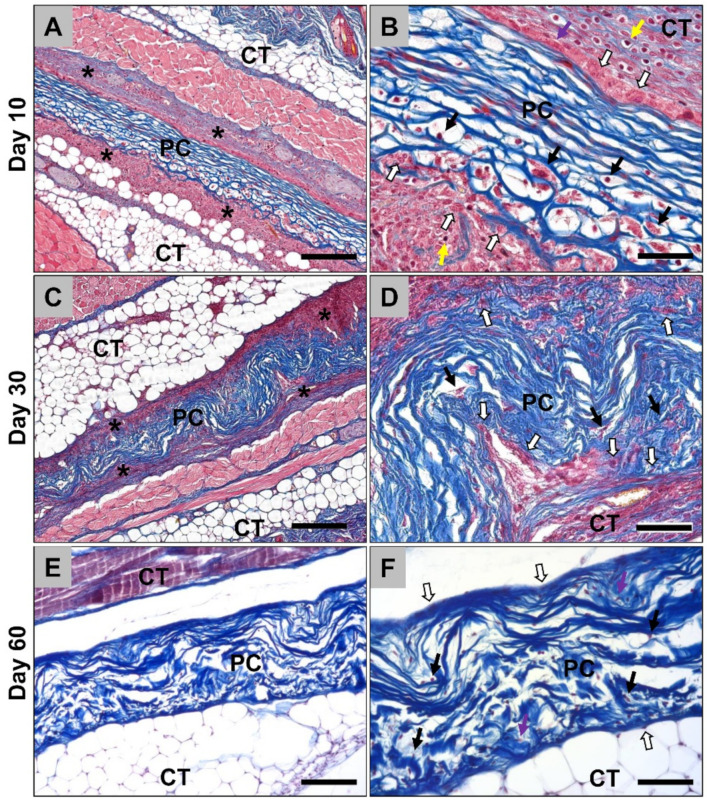
Representative histological images of the porcine pericardium (PC) matrices within the subcutaneous tissue (CT) from day 10 up to day 60 post implantation. (**A**) The membrane (PC) was detectable with the connective tissue (CT) surrounded by a cellular multilayer (asterisks) at day 10 *post implantationem* (Azan-staining, 10× magnification, scale bar = 200 µm). (**B**) A layer of mononuclear cells including mainly macrophages (white arrows) beside single fibroblasts (purple arrows) and lymphocytes (yellow arrows) were observed at the surface of the matrices at this early time point. Only a few mononuclear cells of the monocyte/macrophage line (black arrows) invaded the matrices (Azan-staining, 40× magnification, scale bar = 50 µm). (**C**) After 30 days, the matrices (PC) were still observable without signs of bioresorption within the connective tissue (CT) still surrounded by a cell multilayer (asterisks) (Azan-staining, 10× magnification, scale bar = 200 µm). (**D**) A distinctive thin layer of macrophages (white arrows) remained at the matrix (PC) surfaces and a slight macrophage ingrowth (black arrows) was observable (Azan-staining, 40× magnification, scale bar = 50 µm). (**E**) At day 60 post implantation, the matrices (PC) were embedded within the connective tissue (CT) without any histological signs of further significant occurrence of phagocytic cells indicating their “natural” character (Azan-staining, 10× magnification, scale bar = 200 µm). (**F**) Only single cells were most often found at the matrix surfaces, which were mainly identifiable as fibroblasts (white arrows). Within the matrices, low numbers of cells, i.e., single macrophages (black arrows) and fibroblasts (purple arrow) were observed at this late study time point (Azan-staining, 40× magnification, scale bar = 50 µm).

In the case of the porcine pericardium matrices, the histological analysis showed that the biomaterial was detectable at day 10 *post implantationem* within the subcutaneous connective tissue (Figure 4A). A thin cell-rich wall was observable at the material surfaces that were mainly composed of macrophages as well as single fibroblasts and granulocytes (Figure 4A and B). No multinucleated giant cells were detected at this study time point. A medium number of cells had also penetrated the matrices which were also mainly assignable to the monocyte/macrophage line (Figure 4B). However, most often, the inner cores of the porcine pericardium matrices were free of cellular ingrowth at this early time point (Figure 4B). At this early time point, no vessels were found within the scaffolds (Figure 4A). At day 30 *post implantationem*, the porcine pericardium matrices were still detectable within their subcutaneous implantation beds and their surfaces were covered by a thin cell- and vessel-rich wall (Figure 4C). This reactive wall was composed of mainly macrophages combined with single fibroblasts (Figure 4D). Some single cells of the macrophage line as well as fibroblasts were also observable within the inner cores of the matrices without any signs of material fragmentation or ingrowth of complex tissue components (Figure 4D). No multinucleated giant cells or any vessel ingrowth were detected at this study time point. At day 60 *post implantationem*, the porcine pericardium matrices were still detectable with the subcutaneous connective tissue without any signs of its fragmentation or severe tissue reactions (Figure 4E). Only single cells and mainly fibroblasts beside single macrophages were located on the outer surfaces of the pericardium matrices (Figure 4F). The matrices were penetrated with low numbers of cells, i.e., mainly fibroblasts and macrophages at this later study timepoint (Figure 4F). No further signs of a bioresorption process such as phagocyting cells were visible and the matrices seemed to be integrated within the connective tissue (Figure 4F). 

**Figure 5:**
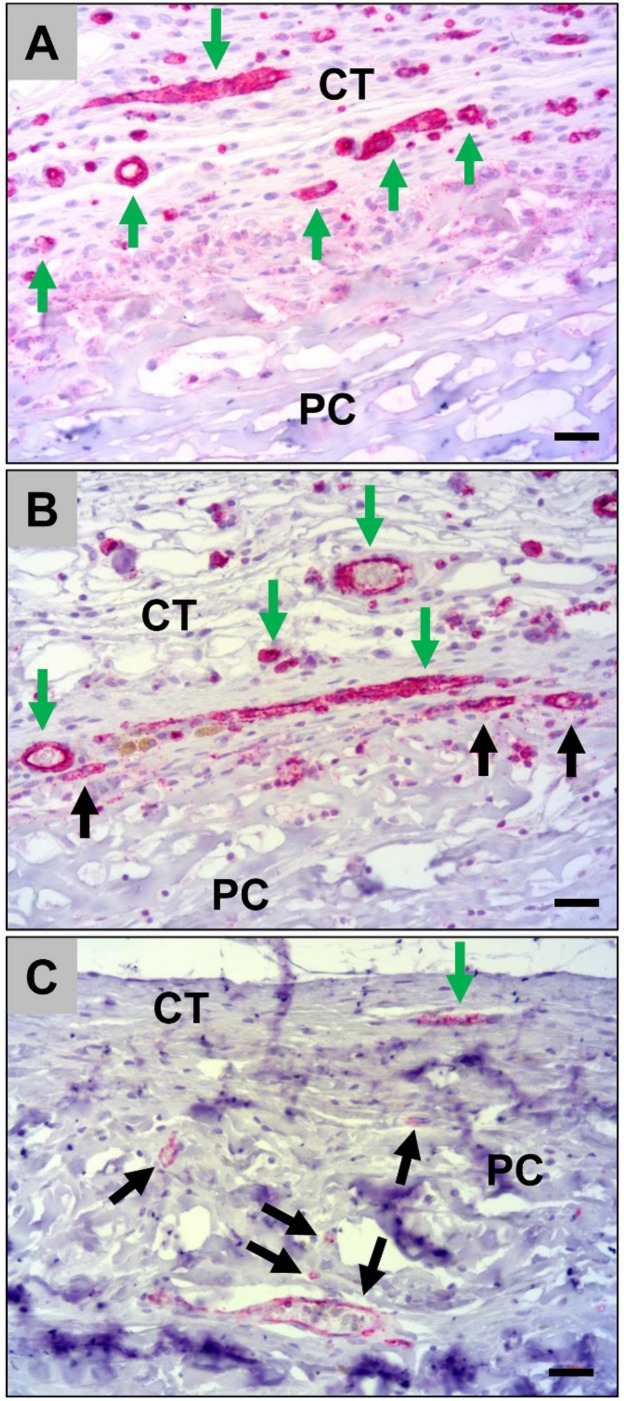
Representative histological images of the vascularization pattern of the porcine pericardium matrices (PC) within the subcutaneous connective tissue (CT) at day 10 (**A**), day 30 (**B**), and day 60 post implantation (**C**). Green arrows = vessels within the surrounding connective tissue, black arrows = vessels within the collagen matrices (CD31-immunostainings, 20× magnifications, scalebars = 20 µm).

Histological analysis of the vascularization of the porcine pericardium matrices showed that high numbers of small and medium-sized vessels were detectable within the material-adherent connective tissue at day 10 *post implantationem* (Figure 5A). At day 30 *post implantationem*, also high numbers of vessels were found within the surrounding connective tissue and single vessels started to invade the porcine pericardium matrices (Figure 5B). At day 60 *post implantationem*, lower numbers of vessels were found in the neighborhood of the matrices compared to day 10 and 30, while moderate numbers of small and medium-sized vessels were detectable within the matrices (Figure 5C). Moreover, the ingrowth of these vessels was not correlated with a tissue ingrowth and the vascular walls were adherent to collagen fibers (Figure 5C).

#### 2.1.2. Histological Analysis of the Immune Response Within the Subcutaneous Tissue

**Figure 6:**
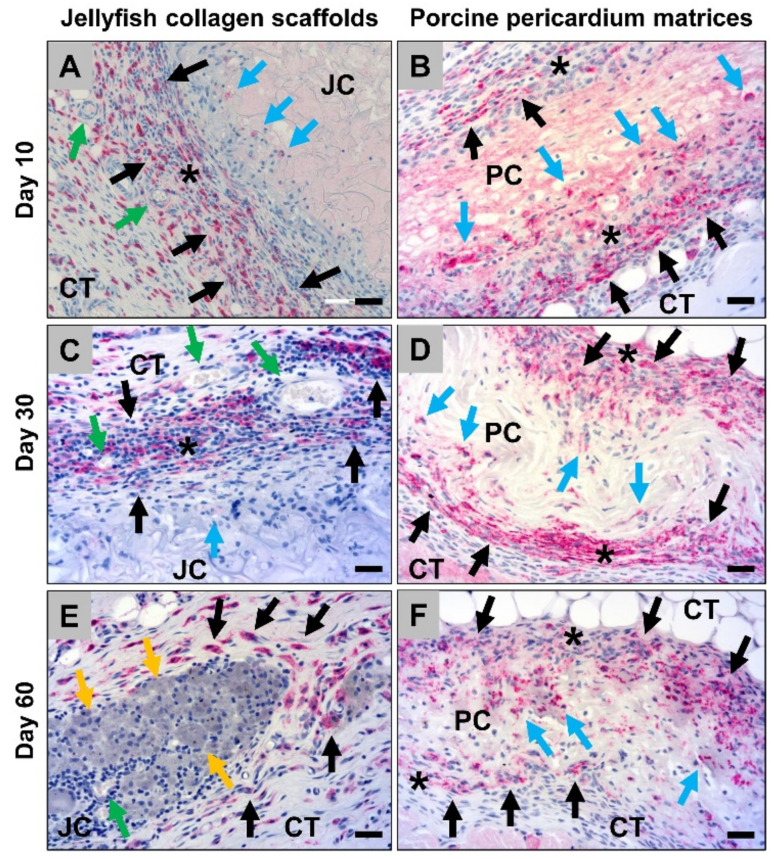
Representative histological images of the anti-inflammatory tissue response to the both analyzed biomaterials, i.e., the jellyfish collagen scaffolds (JC) and the porcine pericardium matrices (PC) within the subcutaneous connective tissue (CT) based in immunohistochemical detection of the CD163-expression at day 10 *post implantationem* (**A**,**B**), day 30 (**C**,**D**) up to day 60 *post implantationem* (**E**,**F**). Black asterisks = cell walls at the material surfaces, black arrows = CD163-positive macrophages within the surrounding connective tissue, blue arrows = CD163-positive macrophages within the biomaterials, green arrows = blood vessels, yellow arrows = CD163-negative macrophages with an increased cytosolic volume (CD163-immunostainings, 20× magnifications, scalebars = 20 µm).

The histological analysis of the occurrence of M2-macrophages showed that high numbers of macrophages within the connective tissue surrounding the jellyfish collagen scaffolds were CD163-positive at day 10 *post implantationem* (Figure 6A). Interestingly, most of the cells, i.e., both the mono- and the multinuclear cells, that invaded the scaffolds were CD163-negative at this early time point (Figure 6A). In addition, in the case of the porcine pericardium scaffolds, most of the macrophages within the adherent connective tissue but also the invaded cells were CD163-positive at this study time point (Figure 6B). At day 30 *post implantationem*, most of the cells within the adherent connective tissue of the jellyfish scaffolds were CD163-positive, while the invaded mono- and multinucleated cells were mostly still CD163-negative (Figure 6C). The analysis, furthermore, showed that nearly all cells within the surrounding connective tissue and the invaded cells within the implant beds of the pericardium scaffolds were CD163-positive at this time point (Figure 6D). At day 60 *post implantationem*, high numbers of the macrophages within the surrounding connective tissue of the jellyfish implantation beds were CD163-positive, while the majority of the cells within the implant bed were negative (Figure 6E). Moreover, most of the cells within the surrounding connective tissue of the porcine pericardium scaffolds as well as most of the invaded macrophages were CD163-positive (Figure 6F).

**Figure 7:**
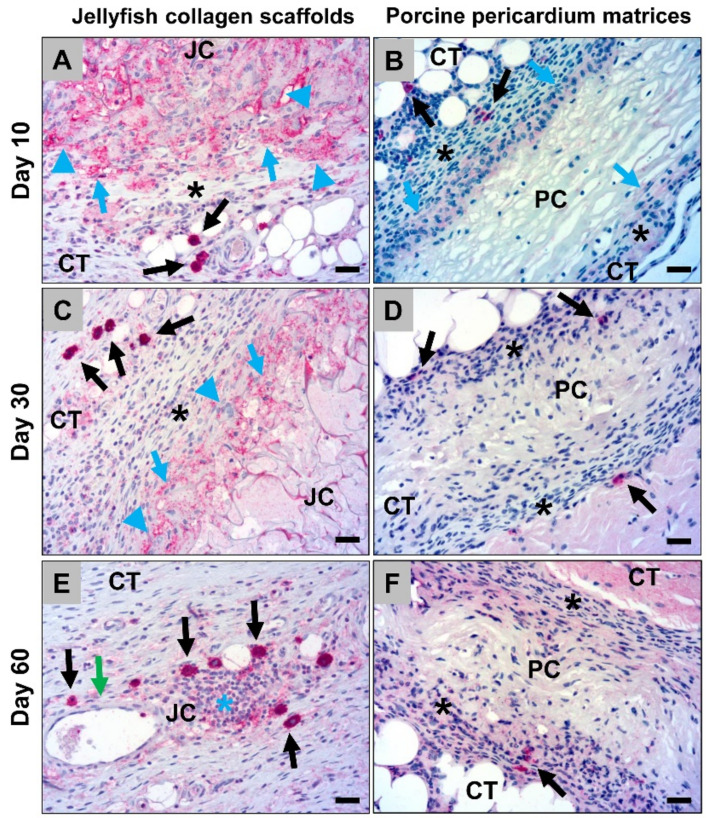
Representative histological images of the pro-inflammatory tissue response to the both analyzed biomaterials, i.e., the jellyfish collagen scaffolds (JC) and the porcine pericardium matrices (PC) within the subcutaneous connective tissue (CT) based in immunohistochemical detection of the CD11c-expression at day 10 (**A**,**B**), day 30 (**C**,**D**) up to day 60 *post implantationem* (**E**,**F**). Black asterisks = cell walls at the material surfaces, blue asterisk = granulation tissue, black arrows = CD11c-positive macrophages within the surrounding connective tissue, blue arrows = CD11c-positive macrophages within the biomaterials (very slight expression), blue arrowheads = CD11c-positive multinucleated giant cells (slight expression), green arrow = blood vessels (CD11c-immunostainings, 20× magnifications, scalebars = 20 µm).

The analysis of the of the occurrence of M1-macrophages revealed that only single CD11c-positive cells were found within the surrounding implant beds of both the jellyfish scaffolds and the porcine pericardium scaffolds at day 10 *post implantationem* (Figure 7A and B). Furthermore, the mono- and multinucleated cells that invaded the periphery of the jellyfish scaffolds showed also a very slight expression of CD11c (Figure 7A). In addition, at day 30 *post implantationem*, only a few macrophages within the adherent connective tissue of the jellyfish scaffolds showed signs of a CD11c-expression and a very slight expression was found in the invaded mono- and multinucleated (Figure 7C). Only single cells within the surrounding connective tissue of the pericardium scaffolds were CD11c-positive at this time point (Figure 7D). At day 60 *post implantationem*, only a few macrophages within the jellyfish implantation beds were CD11c-positive (Figure 7E). In addition, low numbers of macrophages within the surrounding connective tissue of the pericardium scaffolds showed signs of a CD11c-expression (Figure 7F).

#### 2.1.3. Histological Analysis of the Calvarian Implantation Beds

**Figure 8:**
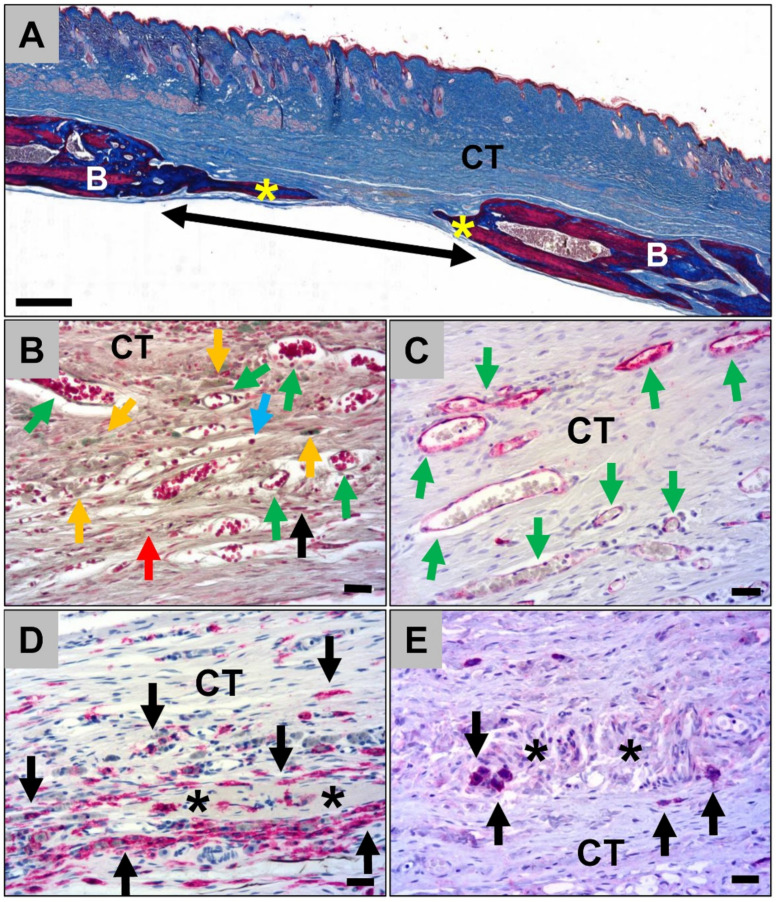
Representative histological images of the bony implantation beds of the jellyfish collagen scaffolds at day 60 *post implantationem*. (**A**) Overview of a bony implantation site (double arrow). Yellow asterisks = newly formed bone, B = local bone, CT = connective tissue (“total scan”, Azan-staining, 10× magnification, scale bar = 1 mm). (**B**) Cell- and vessel-rich connective tissue (CT) within the calvarial defects. Orange arrows = macrophages with an increased cytosolic volume, black arrows = macrophages, red arrows = fibroblasts, blue arrows = lymphocytes, green arrows = blood vessels (Movat’s Pentachome-staining, 20× magnification, scale bar = 20 µm). (**C**) Vascularization pattern (green arrows = blood vessels) of the connective tissue within the calvarial defects (CD31-immunostaining, 20× magnification, scalebar = 20 µm). (**D**) Immunohistochemical detection of anti-inflammatory macrophages (black arrows). Asterisks = remnants of the jellyfish collagen scaffolds, CT = connective tissue (CD163-immunostainings, 20× magnifications, scalebars = 20 µm). (**E**) Immunohistochemical detection of pro-inflammatory macrophages (black arrows). Asterisks = remnants of the jellyfish collagen scaffolds, CT = connective tissue (CD11c-immunostainings, 20× magnifications, scalebars = 20 µm).

Histological analysis of the calvarial defects that were implanted with the jellyfish collagen scaffolds showed that these were nearly completed degraded at 60 days *post implantationem* (Figure 8A). However, moderate bone regeneration could be observed (Figure 8A). Some remnants of the former collagen scaffolds were detectable, which were interestingly associated with moderate numbers of macrophages which showed comparable signs of an increased cytosolic volume as also found in the subcutaneous implantation beds (Figure 8B). Furthermore, the scaffolds were replaced by a cell- and vessel-rich connective tissue including mainly macrophages beside lower numbers of lymphocytes, granulocytes, and fibroblasts (Figure 8B and C). Additionally, the histological analysis of the occurrence of M1- and M2-macrophages showed that most of the macrophages within the calvarial implantation beds were CD163-positive, most often surrounding the remnants of the jellyfish collagen scaffolds (Figure 8D). Only single macrophages, which were most often located within the collagen remnants, showed signs of an CD11c-expression (Figure 8E).

In the control group without biomaterial insertion, low bone tissue regeneration was detectable outgoing from the defect borders. Mainly fibroblasts and macrophages in concert with some single vessels were observable within the defect areas. Furthermore, a more pronounced fibrosis was observed within the calvarial defects of the control group. Moreover, the histopathological analysis of the immunohistochemically stained sections revealed that lower numbers of CD163-positive macrophages were found in the control group compared to the group of the jellyfish collagen scaffolds. In contrast, comparable numbers of CD163-positive macrophages were detectable in both groups.

### 2.2. Histomorphometrical (Quantitative) Analysis 

#### 2.2.1. Analysis of the Immune Response Within the Subcutaneous Tissue

**Figure 9:**
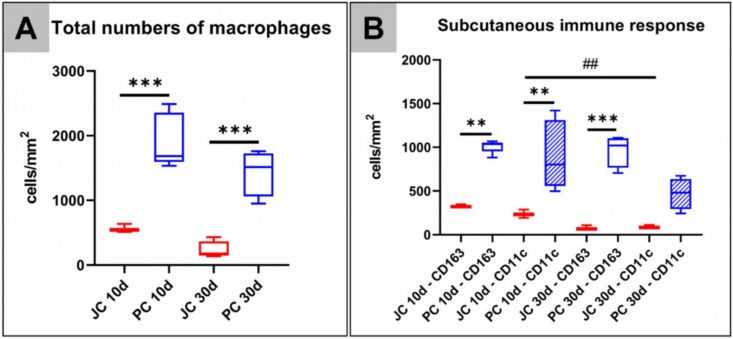
Results of the histomorphometrical analysis of the (**A**) total occurrence of macrophages and (**B**) of the CD163- and CD11c-positive macrophages after implantation of both biomaterials within the subcutaneous connective tissue (* = interindividual significances, # = intraindividual significances).

At day 10 *post implantationem*, the histomorphometrical analysis revealed that significantly higher numbers of macrophages (***p < 0.001) were found within the implant beds of the porcine pericardium matrices (1918.0 ± 415.8 cells/mm^2^) compared to the respective numbers in the implant beds of the jellyfish collagen scaffolds (564.8 ± 64.01 cells/mm^2^) (Figure 9A). In addition, a significant difference in the numbers of macrophages (***p < 0.001) within the implantation beds of the jellyfish collagen scaffolds (230.8 ± 415.8 cells/mm^2^) and the porcine pericardium matrices (1434.3 ± 355.8 cells/mm^2^) was found at day 30 *post implantationem* (Figure 9A).

Moreover, the histomorphometrical analysis of the pro- and anti-inflammatory macrophage subtypes showed that significantly higher numbers of both M1- and M2-macrophages (**p < 0.01) were found within the implant beds of the porcine pericardium matrices (CD163-positive cells: 1010.3 ± 72.80 cells/mm^2,^ CD11c-positive cells: 907.8 ± 391.6 cells/mm^2^) in comparison to the respective numbers within the implantation beds of the jellyfish collagen scaffolds (CD163-positive cells: 327.2 ± 19.62 cells/mm^2^, CD11c-positive cells: 237.6 ± 47.79 cells/mm^2^) at day 10 *post implantationem* (Figure 9B). At day 30 *post implantationem*, only the number of anti-inflammatory macrophages were significantly higher (***p < 0.001) in the group of the porcine pericardium matrices (CD163-positive cells: 963.9 ± 184.7 cells/mm^2^) compared to this subtype in the group of the jellyfish collagen scaffolds (CD163-positive cells: 78.97 ± 26.96 cells/mm^2^) (Figure 9B). No significant differences were found between the numbers of CD11c-positive cells in the group of the porcine pericardium matrices (CD11c-positive cells: 470.3 ± 178.9 cells/mm^2^) and of the jellyfish collagen scaffolds (CD11c-positive cells: 237.6 ± 47.79 cells/mm^2^) at this time point (Figure 9B).

Altogether, the analysis revealed comparable numbers of both macrophage subtypes in the implant bed of both biomaterials but significantly higher pro- and anti-inflammatory macrophage numbers in the group of the porcine pericardium matrices (Figure 9B). 

A significant decrease in occurrence of pro-inflammatory CD11c-positive M1-macrophages was detected in the implantation beds of the jellyfish scaffold between day 10 and 30 (^##^*p* < 0.01), however no significant difference was observed for anti-inflammatory M2-macrophages. 

#### 2.2.2. Analysis of the Level of Bone Regeneration in the Calvarian Implantation Beds

**Figure 10:**
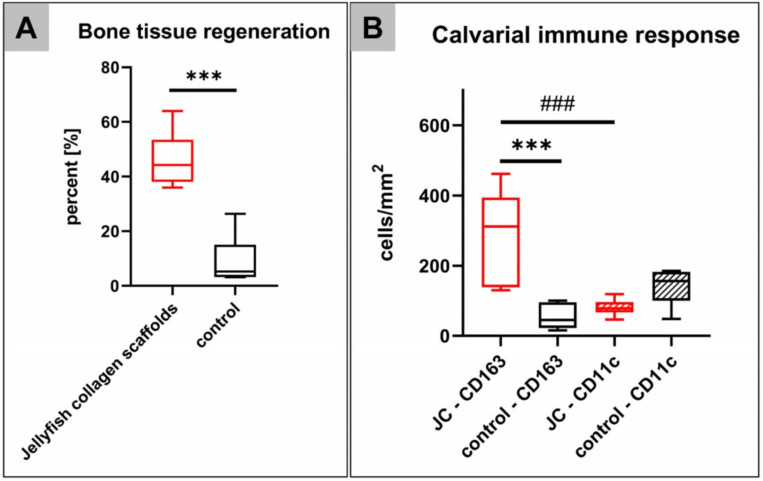
Results of the histomorphometrical analysis of (**A**) the bony regeneration and (**B**) the occurrence of CD163- and CD11c-positive macrophages within the cavarial bone defects (* = interindividual significances, # = intraindividual significances).

An average of 46.20 ± 10.54% de novo bone formation was measured within the calvarial implantation beds of the jellyfish collagen scaffolds at day 60 *post implantationem*. A significant lower average of 9.08 ± 8.97% of newly formed bone was found in the control group without biomaterial insertion (***p < 0.001) (Figure 10A). 

#### 2.2.3. Analysis of the Occurrence of M1- and M2-Macrophages Within the Calvarian Implantation Beds

The histomorphometrical analysis of the occurrence of pro- and anti-inflammatory macrophages within the calvarial implantation beds of the jellyfish collagen scaffolds showed significantly higher numbers of CD163-positive macrophages (288.2 ± 130.3 cells/mm^2^) than CD11c-positive M1 macrophages (80.7 ± 23.3 cells/mm^2^) at 60 days *post implantationem* (^###^ *p* < 0.01) (Figure 10B). Furthermore, significantly higher CD163-positive macrophages were found in this group compared to the control group (54.15 ± 35.24 cells/mm^2^) (***p < 0.001), while no differences between the numbers of CD11c-positive macrophages in both study groups were found (control group: 144.1 ± 55.59 cells/mm^2^) (Figure 10B).

## 3. Discussion

Collagen is a naturally occurring biopolymer that constitutes the major protein of the extracellular matrix of the human organism. Collagen is also an extensively utilized material for tissue regeneration and tissue engineering [17]. For medical applications, collagen is most often extracted from mammalian organisms but it is known that these can often be associated with a pathological risk for different transmitted diseases [17]. While these issues have widely been analyzed and excluded, the purification processes of collagen to remove potential immunogenic components without structure alteration is very challenging [17]. Last but not least, mammalian collagen is increasingly rejected for religious or ethical reasons and the related high production costs [9]. 

Hence, collagen from a non-mammalian origin, seems to be an exciting alternative to address the issues related to the existing collagen-based biomaterials [17]. Jellyfish collagen is generating increasing interest in the field of tissue regeneration and tissue engineering. 

Jellyfish appeared on the evolutionary tree of life about 600 million years ago and has a simple physiology and very low immunogenicity [35]. Its collagen has only 29 non-coding miRNA sequences (microRNA), whereas there are 400 for porcine and bovine [35]. More interestingly, jellyfish collagen has a collagen content of more than 60%, which is shows homology to the mammalian collagen types I, II, and V, which allows the presumption that it can be defined as “collagen type 0” [35]. Song et al. stated that the most abundant amino acid in jellyfish and calf skin collagen is glycine. However, they detected that jellyfish collagen were relatively high in glutamine and alanine but had a lower proline content than calf-skin collagen. Additionally, the amino acid cysteine was present in jellyfish collagen but absent in calf-skin collagen [10]. These findings were recently confirmed by Cheng and colleagues as they analyzed and compared the amino acid composition of jellyfish collagen and calf-skin collagen. They found that the contents of alanine, asparagine, glutamine, and arginine were higher compared to the calf-skin collagen. Specifically, the content of lysine was increased compared to the calf skin type I collagen. The authors concluded that jellyfish collagen can be classified as type I collagen similar to calf-skin collagen with some slight differences between the amino acid composition [36]. 

An increasing global populations of jellyfish is causing a major ecological problem for fin fish, as well being damaging to tourism, near shore power stations, and for off-show aquaculture [9]. The explosion of jellyfish population blooms over recent decades has in part been caused by overfishing the world’s oceans, leading to a lack of natural predators, thus providing the opportunity to explore this as a sustainable alternative to mammalian collagen for tissue engineering applications [9]. Furthermore, jellyfish collagen provides different advantages such as its naturally high batch-to-batch producibility and its reasonable average costs [36].

In this context, the usability of collagen from *Rhizostoma pulmo* (*R. pulmo*) in contrast to other Mediterranean jellyfish species has been revealed due to its comparable biological and cytotoxicity responses to mammalian collagen [22]. Moreover, it has been concluded that jellyfish collagen induces a higher cell viability of fibroblasts and especially of osteoblasts compared to bovine collagen [10,22]. 

Although these study results indicate the usability of jellyfish collagen from *R. pulmo* for tissue regeneration applications, no in vivo studies regarding its immune response and its bone regeneration capacity have been performed up to date. Recent studies have shown that inflammatory mechanisms activate the processes of tissue repair and regeneration [30,37]. Macrophages and multinucleated giant cells (MNGCs) have shown to be key elements of the tissue reaction to a biomaterial and their reactivity seems mainly to “decide” upon the fate of the material and the material-related healing process [30,38,39,40]. In this context, macrophages are able to clear the biomaterial through phagocytosis, control angiogenesis, and extracellular matrix (ECM) remodeling, and regulate inflammation. Macrophages are therefore essential for proper tissue repair and recovery of homeostasis [30], as they are able to express both pro- and anti-inflammatory molecules [40,41]. Thus, it is of great importance to analyze the immune response to a biomaterial before its clinical application to ensure its safety and biocompatibility. On this account, two cumulative in vivo studies using established implantation models, i.e., the subcutaneous and the calvarian implantation model, in Wistar rats and previously published histological, histopathological, and histomorphometrical methods have been used to analyze the tissue reactions to jellyfish collagen scaffolds [31,42,43,44]. For the subcutaneous implantation model, a collagen matrix originating from porcine pericardium was used as a control material as it has been stated to be fully biocompatible for dental applications [32,33,34].

Initially, the implantation study showed that the implanted jellyfish collagen scaffolds (Jellagen^®^-3D scaffolds manufactured by Jellagen^®^ Ltd., Cardiff, UK) were detectable within the subcutaneous connective tissue up to 60 days post implantation, while the main degradation process took place between 10 and 30 days. At 60 days *post implantationem*, only remnants of the former jellyfish collagen scaffolds were observable, resulting in a cell- and vessel-rich connective tissue. Thereby, the jellyfish collagen scaffolds were gradually degraded from their periphery towards the scaffold center as also observed in the case of other biomaterials such as injectable bone substitute materials [45,46,47,48]. In comparison, the matrices based on porcine pericardium were stable up to 60 days, and interestingly, the inflammatory response associated with these matrices at day 10 and 30 was nearly completely decreased up to this latest study time point. It can therefore be assumed that these varying integration patterns are induced by the differences in the collagen types and their related sources. As described, jellyfish collagen mostly resembles Type I collagen, which is also the main collagen type within the mammalian skin [23,36]. In this context, it is well known that even collagen materials based on mammalian skin are rapidly degraded [49,50]. To increase collagen stability, cross-linking using different chemical agents has been robustly used as strategy to prolong the standing time of collagen materials from this source [51,52]. However, it has been shown that the cross-linking of collagen materials often leads to a stronger inflammatory tissue response due to a rapid material degradation and thus producing material incompatibilities with undesirable tissue responses that are dependent on the kind of cross-linking agents used [53,54]. Interestingly, the analyzed jellyfish collagen-based scaffolds use in this experiment were crosslinked by carbodiimide treatment, EDC (1-ethyl-3-(3-dimethylaminopropyl-carbodiimide hydrochloride) in the presence of NHS (N-hydroxy-succinimide), which constitutes one of the most successful chemical crosslinking treatments for collagen-based biomaterials [55,56]. This treatment method was shown to allow the production of nontoxic collagen materials as both EDC and NHS are not taking part in the linkage process and the resultant by-products can be easily removed by washing [57]. 

Interestingly, the porcine pericardium is also mainly composed of type I collagen and has been widely used as a raw material for the fabrication of biomaterials used in various areas of health sciences [58]. In contrast to the analyzed jellyfish collagen scaffolds, the porcine pericardium has a special collagen fiber orientation due to its special functionality as a dominant load bearing component [59]. Moreover, the mammalian pericardium contains highly organized collagen fibers and bears a higher extent of natural crosslinking based on a higher glycosaminoglycan (GAG) content which makes this tissue denser compared to other tissues [60,61]. Altogether, it is assumable that both the collagen composition and also the crosslinking treatment of the jellyfish collagen scaffolds could be the basis for the observed faster degradation in concert with the occurrence of multinucleated giant cells, which are fused macrophages that are supposed to have a higher phagocytosis capacity [62,63].

The described integration behavior providing a gradual cell invasion and degradation has also shown to optimally interact with the bony integration process in the case of different bone pastes [45,46,47]. Bone regenerative properties of bone pastes are proved in both preclinical and clinical studies and it could thus be assumed that the jellyfish collagen scaffolds may be suitable for the same application [45,46,47]. However, it was described that the bone pastes serve as a scaffold-like structure up to 6 months after implantation. In contrast to the analyzed collagen scaffolds, this observation is even based on the inclusion of natural or synthetic bone substitute granules that are composed of calcium phosphates, while the included hydrogel components were also rapidly degraded. Thus, it is assumable that the jellyfish collagen scaffolds may be degraded too fast to act as an osteoconductive scaffold structure based on the observed degradation behavior of the jellyfish collagen scaffolds within the subcutaneous connective tissue. However, the results observed by the subcutaneous implantation lead to the conclusion that the resulting vessel-rich connective tissue observed at 60 days post implantation may provide an optimal basis for bone tissue regeneration as also shown in the case of other collagen-based materials [64,65]. In this context, it is well known that the bone regeneration process is optimally supported by a sufficient vascularization that is able to guarantee effective nutrient supply and waste removal to individual cells [17]. Thus, the highly vascularized tissue after degradation of the jellyfish collagen scaffolds might be a basis for optimal material-mediated bone regeneration, as blood vessels also supply the skeleton with specific hormones and growth factors secreted by other tissues stimulating the osteoblastic activity [66].

Furthermore, the second in vivo study that was conducted, evaluated the impact of implantation of the jellyfish scaffolds into bone defects to enable confirmation of this suspicion. The calvarial implantation of the jellyfish scaffolds also ended in a highly vascularized connective tissue after 60 days. Interestingly, the histomorphometrical results revealed an average of 46.20% de novo bone formation that was significantly higher compared to the control group. Comparable studies reported, for example, approximately 35% bone formation in case of collagen/chitosan scaffolds at 8 weeks after implantation as well as of 55% and 46% in case of collagen Type I gel and laminin at 3 months [67]. Moreover, different other studies showed lower or comparable bone healing capacities for collagen-based composite scaffolds with bioceramic components [68]. In this context, the potential use of collagen in wound repair and its main therapeutical applications such as burns, urological surgery, gynecological surgery, dentistry and oral surgery, reconstructive surgery, abdominal and vascular surgery, and orthopedy are well known [69]. The mechanisms of collagen in wound repair are described with particular emphasis on its hemostatic effect, its interaction with platelets and fibronectin, and its cellular influence onto macrophage polarization and the “scaffold” role for fibroblastic proliferation [69]. The rate of newly formed bone tissue—in concert with the data from the subcutaneous implantation study part—in the present study, thus shows the excellent biocompatibility of the jellyfish collagen scaffolds and its (bone) tissue healing properties.

The analysis of the immune responses to the jellyfish collagen scaffolds interestingly revealed significantly lower numbers of macrophages within the subcutaneous implant beds of the jellyfish collagen scaffolds compared to the respective numbers in the implant beds of the porcine pericardium matrices at both, day 10 and 30 post implantation. This first result indicates an overall weaker immune response to jellyfish collagen than to porcine pericardium matrices. Interestingly, this result is not supported by previous studies that concluded that the jellyfish collagen scaffolds induce an immune response comparable with bovine collagen or gelatin [10]. However, the analysis of the occurrence of pro- and anti-inflammatory macrophages within the subcutaneous implant beds of both materials revealed comparable numbers of both macrophage subtypes but significantly higher pro- and anti-inflammatory macrophage numbers in the group of the porcine pericardium matrices. 

The histomorphometrical analysis revealed significantly higher numbers of both, pro- and anti-inflammatory, macrophage subtypes within the implant beds of the porcine pericardium matrices in comparison to the respective numbers within the implantation beds of the jellyfish collagen scaffolds at day 10 post implantation. However, at day 30 post implantation, only the numbers of anti-inflammatory macrophages were significantly higher in the group of the pericardium-based materials, while no differences of the occurrence of pro-inflammatory cells were found in both study groups. Thus, both biomaterials induced balanced numbers of both macrophage subtypes, which supports their good biocompatibility. In this context, it is additionally believed that a biomaterial should not induce a severe inflammatory tissue response, which would be indicated by significantly higher numbers of pro-inflammatory macrophages [27,29,38]. It is also understood that a biomaterial should evoke an overall anti-inflammatory cellular response to optimally support the process of (bone) tissue regeneration [27,29,38]. However, it has also been shown that even bioresorbable biomaterials such as both natural and synthetic bone substitute materials induce pro-inflammatory cells that express multiple enzymes such as tartrate-resistant acid phosphatase which are involved in biodegradation processes [39,70,71,72]. In case of bone substitutes, it has been assumed that pro-inflammatory cells are thus indispensable for the degradation of the materials [39]. It has furthermore been shown that macrophages polarized into the M1 subtype by the exposure to Toll-like receptor ligands or Th1 cytokines, increases the expression of pro-inflammatory cytokines and the production of reactive oxygen species (ROS) [73]. In this context, it has been shown by Al-Maawi and colleagues that a collagen-based barrier membrane also induced mono- and multinucleated phagocytes expressing the TRAP molecule [74]. Altogether, these results lead to the conclusion that pro-inflammatory cells are also a key component of biodegradable collagen-based biomaterials that “help” to facilitate their functionality and biocompatibility up to the condition of a restitutio ad integrum. The results of the present study seem to underline this assumption, but further studies are needed to clarify these biological processes onto the molecular level. However, the present study had limitations, which led to limited information about the degree of the inflammation response that was solved by the counting of cells. In this context, the quantification of the expression of pro-and anti-inflammatory molecules might lead to further clarification of the tissue responses induced by jellyfish collagen biomaterials. For example, more complex methods, such as laser-assisted cell microdissection, which allows for the measurement of cytokine release from single cells or cell types, are needed for the examination of the cytokine levels released from biomaterial-induced macrophages and BMGCs. 

Interestingly, the results revealed the long-term anti-inflammatory response to the jellyfish collagen scaffolds as a significant decrease in occurrence and detection of pro-inflammatory macrophages in the implantation beds of the jellyfish scaffold between day 10 and 30. This observation is underlined by the fact, that significantly higher numbers of anti-inflammatory macrophages were detected within the calvarial implantation beds of the scaffolds within their bony implantation beds at 60 days post implantation. This result was also confirmed by the significantly higher numbers of anti-inflammatory cells in the jellyfish scaffolds group compared to the control group in correlation with the significantly higher amounts of newly formed bone.

The question arises why the jellyfish collagen scaffolds induced a more pronounced anti-inflammatory tissue response. This could be related to both the amino acid sequences and secondary structures of the jellyfish collagen. The secondary structure of all collagens is a right-handed triple helix of three polypeptide α-chains derived from the repeating amino acid sequence (Gly-X-Y)_n_, where X and Y can be any amino acid [75]. The helical conformation of jellyfish collagen was confirmed by Miki et al. [76]. Proline was detected to be often located at positions X and Y, restricting the dihedral angle of the main chain, and hence contributing to triple helical conformation. Fewer proline residues were observed to be post-translationally modified to hydroxyproline in jellyfish collagen than in fish scale and pig skin. A low hydroxyproline content is associated with a decrease in thermal stability, which can be observed for jellyfish collagen. The native temperature of this organism might be a possible explanation for this phenomenon [76]. Cheng et al. concluded that the amino acid composition differences between jellyfish and typical type I collagen lead to structural changes of jellyfish collagen [36]. In context of the present study, it can be assumed that the structural changes have led to the observed immune response differences. It is already known that proteins have major impact onto different cellular responses such as also described for the so-called “Vroman effect” [77,78]. This effect describes the adsorption of proteins such as albumin, fibrinogen, fibronectin, vitronectin, and gammaglobulins, as well as lipids, sugars, and ions, on the implant surface influenced by the specific characteristics of the biomaterial surface (such as energy, chemistry, topography, and roughness) [77,78]. Thus, the specific biomaterial surface topography influences the type, the amount, the composition, and the conformation changes of molecules [77]. This material-specific molecule composition leads to the adhesion of different cell types such as monocytes or macrophages and a material-specific expression pattern of signal molecules by these cells [28,38,77]. Consequently, it is assumable that the described structural changes in combination with the different chemical alterations due to the diverging amino acid content induced the observed responses to the jellyfish collagen scaffolds. These findings also underline the observation of the importance of the molecule layer onto the material surface as crucial determinant of the overall tissue reaction to a biomaterial [28,77].

Another explanation for the observed degradation behavior is the already described differences in the thermal stability of jellyfish collagen by Miki et al. [76]. This study revealed that the jellyfish collagen showed a high solubility at neutral pH was quite high. Thus, it is also assumable that the process of scaffold degradation takes place via dissolution in concert with the cell-mediated degradation as already known from calcium-phosphate-based bone substitute materials [79]. However, further studies have to clarify both the consequences of the differences of the amino acid content and related immune responses, as well as the dissolution behavior of jellyfish scaffolds.

Altogether, the presented study results show that the tissue response to the jellyfish collagen scaffolds includes a long-term anti-inflammatory macrophage response and an optimal vascularization pattern within their implant beds. Together with the directed integration behavior that has shown to be in accordance with the concept of guided bone regeneration (GBR), which includes directed growth of tissues and new bone at sites with insufficient volumes or dimensions of bone for proper function and restoration, seems to pave the way for successful (bone) tissue regeneration. It is assumable that this integration pattern is also perfectly suitable for different other clinical indications. Moreover, the results showed that all analyzed materials are biocompatible.

## 4. Materials and Methods

### 4.1. Biomaterials

#### 4.1.1. Jellyfish Collagen Scaffolds

The analyzed 3D scaffolds (Jellagen^®^-3D scaffolds) are composed of highly purified jellyfish collagen originating from the jellyfish species *Rhizostoma pulmo* (sourced from Jellagen^®^ Limited, Cardiff, UK). The scaffold is cross-linked to increase mechanical strength. According to the manufacturer Jellagen Ltd., the Jellagen^®^-3D scaffold is crosslinked by carbodiimide treatment, EDC (1-ethyl-3-(3-dimethylaminopropyl-carbodiimide hydrochloride) in the presence of NHS (N-hydroxy-succinimide) (EDC: 25 mM; NHS: 6 mM) dissolved in 95% ethanol for 12 h at 4 °C to achieve a final 1%-EDC cross-linked collagen matrix scaffold having an average pore size of c100 µm. After cross-linking, the sponges were washed five times by deionized water and lyophilized. The scaffold is intended for universal applications in regenerative medicine.

#### 4.1.2. Porcine Pericardium-Based Collagen Matrices 

As a control material, newly developed collagen matrices originating from porcine pericardium (botiss biomaterials GmbH, Zossen, Germany) were used. The biomaterials have an increased content of type III collagen. The purification process includes an initial wet-chemical treatment followed by lyophilization, and sterilization via ethylene oxide gas. Finally, the pericardium matrices show a porous 3D structure. 

### 4.2. In Vivo Study Design, Implantation, and Explanation Procedure

The described in vivo experiments were authorized by the Ethical Committee of the Faculty of Medicine (University of Nilš, Serbia), based on the decision number 323-07-00073, on date: 2017-05/7 of the Veterinary Directorate of the Ministry of Agriculture, Forestry and Water Management of the Republic of Serbia. The experiments were performed at the Faculty of Medicine (University of Niš, Serbia), as well as the animal housing. All animals were kept under standard conditions, such as artificial light, water ad libitum, and regular rat pellet. Standard pre- and postoperative care was ensured. 

#### 4.2.1. Subcutaneous Implantation and Explanation Procedure

The biomaterials were implanted subcutaneously in 38 male Wistar rats. The rats were 10–12 weeks old and were obtained from the Military Medical Academy (Belgrade, Serbia). The animals were randomly divided into two experimental groups. The first group was implanted with the jellyfish scaffolds (n = 19 animals in total) and the second group was implanted with porcine collagen matrices (n = 19 animals in total). The subcutaneous implantation was performed following the protocol described by Barbeck et al. [39,72,80,81,82]. In short, the animals were first sedated via an intraperitoneal injection (10 mL ketamine (50 mg/mL) with 1.6 mL Xylazine (2%)), shaved and disinfected, before introducing an incision in the rostral portion of the interscapular region and biomaterials implantations into preformed subcutaneous pockets. Subsequently, the wounds were sutured. 

The implantation area, together with the peri-implant tissue, was explanted at 10, 30, 60, or 90 days after implantation, in 4, 5, 5, and 5 rats respectively in each group. The animals had been euthanized with euthasol (400 mg/mL). The explanted tissues were fixed in a 4% formalin solution for 48 h and were then stored in PBS until they were treated by a histological workup process. 

#### 4.2.2. Calvarial Implantation

**Figure 11:**
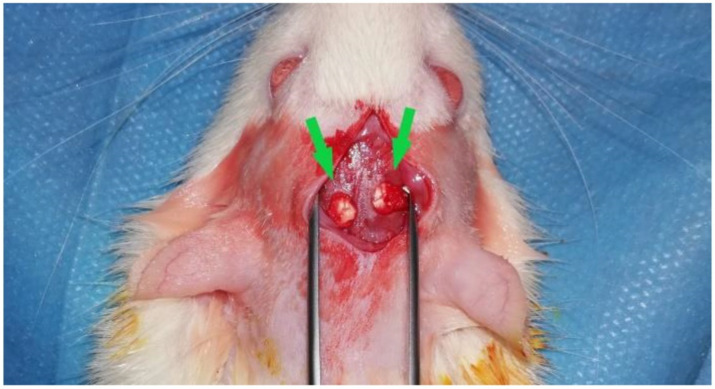
Representative picture of the implantation procedure. Two calvarian defects were implanted with jellyfish collagen scaffolds (green arrows).

The scaffolds were implanted in calvarial defects on each skull hemisphere of 4 male Wistar rats (n = 8 defects). Additionally, 3 rats (n = 6 defects) were used as the control group, conducting the surgery without biomaterial implantation. The rats were 10–12 weeks old and were obtained from the Military Medical Academy (Belgrade, Serbia). The calvarial implantation was performed following the protocol described by Sieger et al. [43]. In short, the animals were first anesthetized via an intraperitoneal injection (10 mL ketamine (50 mg/mL) with 1.6 mL Xylazine (2%)), shaved and disinfected, before introducing an incision in the skin and muscle tissue covering the calvaria. Two defects per calvaria with a diameter of 5 mm were created with a trephine bur, under local anesthesia with lidocaine (2%) and constant sterile saline irrigation. Subsequently, the trephined calvarial bone was removed followed by biomaterial implantation into each bone defect (Figure 11). In the control group, no biomaterial was implanted in the bone defects. Afterwards, the wounds were closed with suture material.

All defect areas, together with the surrounding tissue, were explanted at 60 days after implantation. Previously, the animals had been euthanized with euthasol (400mg/mL). The explanted tissues were fixed by placing them in a 4% formalin solution for 48 h and were then stored in PBS until further histological preparations.

### 4.3. Sample Preparation and Staining Procedures

The tissue explants were divided in the center of the implantation area into two even segments of identical dimensions. Decalcification over 14 days was necessary to process the calvaria samples and was conducted with decalcifier soft (Carl Roth GmbH, Karlsruhe, Germany). Dehydration (MTP carousel tissue processor, SLEE, Mainz, Germany) in a series of increasing alcohol concentrations (80%, 96%, 100%), xylol exposure and hot liquid paraffine (Paraplast plus, McCormick Scientific, St. Louis, MO, USA) was performed before embedding with orientation on cutting edges. Slides of a thickness of 3–5 μm were prepared by means of a rotation microtome (SLEE, Mainz, Germany). Three slides of every tissue explant were used for histochemical staining (Hematoxylin and Eosin (H&E), Movat’s pentachrome and Masson’s trichrome). The staining was performed following previously described methods [44,45,83]. 

Three further slides of every explant were used for immunohistochemical detection as previously published [31,42,43,44]. M1- and M2-macrophage subforms were detected by means of antibodies against the pro- and anti-inflammatory molecules integrin alpha x (CD11c) (abx231412, Abbexa Ltd., Milton, United Kingdom) and hemoglobin scavenger receptor (CD163) (ab182422, abcam, Cambridge, United Kingdom). Additionally, vascularization was detected by means of antibody against platelet endothelial cell adhesion molecule (CD31/PECAM-1) (ab182981, abcam, Cambridge, United Kingdom). In short, after dewaxing in xylol and rehydration in a decreasing series of alcohol (80%, 96%, 100%), slides for all antibodies were initially pre-treated with TRIS-EDTA pH 9 for 15 min at 150 °C in a steamer, followed by 35 min cooling down in pretreatment buffer and equilibration using cold wash buffer (Wash Buffer 20×, Zytomed Systems, Berlin, Germany). Subsequently, slides were prepared with protein blocking solution (Blocking Solution, Zytomed Systems, Berlin Germany) for 10 min. After incubation with the respective primary antibodies at room temperature for 40 min was conducted, it was followed by incubation with the biotinylated secondary antibody (ZytoChem Plus AP-Kit, Zytomed Systems, Berlin, Germany) for 15 min at room temperature. Detection of the antigen was performed using streptavidin–alcaline–phosphatase conjugate (Zytomed Systems, Berlin, Germany) and permanent AP-red chromogen (Zytomed Systems, Berlin Germany). Counterstaining was performed using Mayer’s hemalum solution (Merck KGaA, Darmstadt, Germany).

### 4.4. Histopathological Analysis

The focus of the qualitative histopathological evaluation was on the tissue reactions to the biomaterial and potential interactions of the biomaterial with newly formed bone as previously described [39,40,72,84]. A light microscope (Axio Scope. A1) combined with an Axiocam 305 color digital camera and connected to a computer system running the ZEN Core software (all: Zeiss, Oberkochen, Germany) was used for the analysis and an established protocol was performed. These analyses included the evaluation of the following parameters: fibrosis, hemorrhage, necrosis, vascularization and the presence of neutrophils, lymphocytes, plasma cells, macrophages, and multinucleated giant cells (BMGCs). 

### 4.5. Histomorphometrical Analysis 

As previously described [31,42,43,44], the areas of interest were digitized with the aid of a specialized scanning microscope, which consists of an Axio Scope. A1 microscope combined with an Axiocam 305 color digital camera and an automatic scanning table (Maerzhaeuser, Wetzlar, Germany) connected to a computer system running the ZEN Core software V3 (all: Zeiss, Oberkochen, Germany). The histomorphometrical analysis included measurements of the occurrence of pro- and anti-inflammatory macrophages within the implant beds of the scaffold and the amounts of regenerated bone. The implantation areas of both the subcutaneous and the bony implant beds were initially measured. For both study parts, the M1- and M2-macrophage subforms were manually counted and the corresponding densities were calculated (cells/mm^2^). For the calvarian study part, the amounts of new bone in the calvarial defects were manually measured (in µm^2^) and related to the defect area, resulting in percentages of newly formed bone. 

### 4.6. Statistical Analysis 

An analysis of variance (ANOVA) was performed via the RStudio Team (2015) (RStudio: Integrated Development for R. RStudio, Inc., Boston, MA, USA) and GraphPad Prism 7.0d software (GraphPad Software Inc., La Jolla, California, USA). Statistical differences were considered as significant if the p-values were below the significance threshold of 0.05 (* *p* ≤ 0.05) and were considered highly significant if the *p*-values were less than 0.01 (** *p* ≤ 0.01) or even less than 0.001 (*** *p* ≤ 0.001). The quantitative data were shown as mean ± standard deviation. 

## 5. Conclusions

The results of the present study show that the Jellagen^®^-3D jellyfish collagen scaffolds are resorbed until day 60 after implantation by stepwise integration within the subcutaneous connective tissue in accordance to the concept of guided bone regeneration (GBR). Interestingly, the degradation process ended in a vessel-rich connective tissue that is supposed to be an optimal basis for (bone) tissue regeneration. The results showed an overall weaker immune response to the jellyfish collagen than to porcine pericardium matrices, as significantly lower numbers of macrophages were observed within the subcutaneous implant beds of the jellyfish collagen scaffolds compared to the respective numbers in the implant beds of the porcine pericardium matrices. However, both collagen-based biomaterials induced balanced numbers of pro- and anti-inflammatory macrophage subtypes, which supports their good biocompatibility. Moreover, the study revealed a long-term anti-inflammatory response to the jellyfish collagen scaffolds in the cavarial implantation beds. A de novo bone formation of 46.20 % was observed in the calvarial implantation beds, which was significantly higher compared to the control group without biomaterial insertion. Overall, the present study thus shows the excellent biocompatibility of the jellyfish collagen scaffolds and show their successful support for the process of (bone) tissue regeneration.
